# Knowledge of risk and protective factors for dementia in older German adults A population-based survey on risk and protective factors for dementia and internet-based brain health interventions

**DOI:** 10.1371/journal.pone.0277037

**Published:** 2022-11-07

**Authors:** Andrea E. Zülke, Melanie Luppa, Sebastian Köhler, Steffi G. Riedel-Heller

**Affiliations:** 1 Faculty of Medicine, Institute of Social Medicine, Occupational Health and Public Health, University of Leipzig, Leipzig, Germany; 2 Department of Psychiatry and Neuropsychology and MHeNS School for Mental Health and Neuroscience, Maastricht University, Maastricht, The Netherlands; Cardiff University, UNITED KINGDOM

## Abstract

**Background:**

Evidence on potentially modifiable risk factors for dementia is accumulating rapidly, including e.g. physical inactivity, hypertension, or diabetes. It is unclear to what extent these risk factors are known among the general population in Germany. We investigated knowledge on risk and protective factors for dementia and openness to eHealth interventions for brain health in the older general population in Germany.

**Methods:**

A population-based telephone survey among randomly selected community-dwelling adults aged ≥ 60 years was conducted. We assessed sociodemographic factors, knowledge on risk and protective factors for dementia, openness towards eHealth and psychosocial outcomes (health literacy, resilience). Factors associated with interest in information on brain health and openness towards eHealth interventions were assessed using multivariable logistic regression.

**Results:**

Of n = 500 respondents (mean age: 74.8 years, % female: 62.8), 67.9% believed that dementia risk is modifiable. Participants mostly endorsed physical and cognitive activity as protective factors and social isolation as a risk factor. Knowledge on cardiovascular risk factors was low to moderate. 38.0% were interested in information on dementia risk reduction. Better knowledge of risk factors for dementia and higher age were linked to interest in information on brain health. Being widowed and higher levels of health literacy were associated with lower interest in information. Openness to eHealth interventions was moderate (46.2%). Younger age, better knowledge of risk and protective factors were linked to openness towards eHealth tools, as was knowing someone with dementia and interest in information on brain health.

**Conclusion:**

Belief in preventability of dementia was higher in our sample than previously reported. However, knowledge on cardiovascular risk factors for disease was insufficient and more information and intervention approaches targeted at older adults are needed. Interest in information on dementia risk reduction and eHealth approaches was moderate, and further studies are warranted to assess needs and concerns of older adults regarding dementia prevention.

## Introduction

More than 55 million people worldwide are currently living with dementia, a number projected to triple until 2050 due to population ageing and increases in life expectancy [1]. Despite extensive research efforts, there is no curative treatment available to date. This has resulted in a growing interest in identifying modifiable risk factors and design of possible pathways for prevention of cognitive decline and dementia [2–4]. Currently, validated modifiable risk factors include low education in early life, hearing loss, traumatic brain injury, hypertension, obesity, excessive alcohol consumption (*>*21 units per week) in midlife, diabetes mellitus, depression, physical inactivity, smoking, social isolation, and exposure to air pollution in later life. Together, these risk factors are estimated to account for approximately 40% of all cases of dementia in high-income-countries, with prevention potential in low- and middle-income countries being even greater [5].

While there is increasing awareness of these risk factors among the scientific community, there seems to be a lack of knowledge on modifiable risk- and protective factors for dementia in the general population. In a review covering 38 surveys from the EU, the US, Eastern Asia, Israel and Australia (total: n = 36.519 individuals), about 50% of respondents stated that dementia was part of the natural ageing process [6]. Over two thirds (70.1%) of respondents stated that dementia was caused by normal ageing in a global survey by Alzheimer’s Disease International [7]. Although belief in preventability of dementia slightly increased over the last years, assumptions that the condition constitutes a normal part of ageing remained rather stable over time [7,8]. Certain risk factors appear to be better known to the general population than others: Presented with established risk factors for dementia, participants endorsed an unhealthy diet, low cognitive and physical activity more often than e.g. cardiovascular diseases [9]. On the other hand, parts of the population endorse lifestyle factors without known impact on dementia risk, e.g. 75% of respondents in two studies conducted in the US believed that regular intake of vitamins was able to prevent dementia [6]. In a German survey on dementia knowledge conducted a decade ago, 55% of respondents expressed belief that dementia could be prevented, which is comparable to international figures [6]. Asked about possible prevention measures participants’ spontaneous answers mainly included cognitive activity, e.g. brain/memory training (47.4%) or general mental activity, e.g. learning a language, reading or writing (33.9%; [10]). Lack of knowledge about diseases and their associated risk factors have been found an important barrier for pursuing a healthy lifestyle in older age [11] and might also complicate help-seeking and early diagnosis for older adults [12,13]. Therefore, it is worthwhile to assess older adults’ knowledge about risk and protective factors for dementia.

Conditions like diabetes, hypertension and obesity are highly prevalent among older adults in Germany (diabetes: 13.8% among adults 60–69, 21.9% among those 70–79 years of age [14]; hypertension: 59.8% in those 60–69 years and 74.2% in those 70 to 79 years old [15]; obesity: 34.8% in women aged 60–69 years, 41.6% in the age group 70–79; 33.1% in men aged 60–69, 31.3% in men aged 70–79 years [16]. Therefore, improved knowledge on and targeted interventions for these risk factors might constitute an effective way of preventing both cardiovascular diseases and reducing risk for dementia.

Research on modifiable lifestyle factors that impact dementia risk is still comparatively recent but evolving rapidly. Therefore, it is reasonable to expect that not all validated risk and protective factors are already known to the general public [17]. Still, these findings point towards a large untapped potential for dementia risk reduction on a population level. Moreover, knowledge on potentially modifiable lifestyle risk and protective factors is unequally distributed within societies., It has been shown that older people (65 years and older) and those with lower levels of education less often believe that cognitive decline and dementia are preventable [9,18,19]. This implies that especially those who are already at increased risk for developing dementia are inadequately informed about the condition and its risk factors.

Regarding information on health-related topics, the internet has gained increased importance not only in younger generations. In a recent study from Germany, 43% of respondents aged 75 years and older reported regular internet usage, 55.7% thereof used the internet for information on health-related questions [20]. People between 60 and 69 years of age constitute the most rapidly growing share of internet users in Germany ([21]), raising the question whether internet-based approaches may be feasible for increasing dementia awareness and promoting brain health. In a recent study of a community-based sample of middle-aged and older adults from the Netherlands, 54% of respondents were interested in using internet-based measures to improve their brain health, with further 27% considering it [9]. Internet-based (or: eHealth) interventions have been highlighted as innovative approaches for delivering tailored advice on brain health, allowing for large-scale implementation of highly individualized interventions [22–24]. Use of self-management tools, e.g. delivered in an eHealth-setting, might further increase adherence to guidelines and recommendations for a healthy lifestyle [25]. Moreover, eHealth interventions can provide information through various channels, e.g. through written and audiovisual formats, which has been shown to increase processing of health-related information and might facilitate inclusion of older adults with varying levels of health literacy [26]. Recently, the Healthy Aging Trough Internet Counselling in the Elderly (HATICE)-trial, conducted across several European countries, provided evidence that a guided eHealth-program can improve health-related lifestyles and reduce risk for several cardiovascular diseases in the elderly general population [27]. At trial completion, a majority of participants stated that the intervention was both an effective and fun way to improve their health [25]. Small to moderate effects of eHealth-interventions on cognitive function and dementia risk scores have been reported in systematic reviews and meta-analyses [24].

To the best of our knowledge, there is currently no study assessing awareness of dementia risk factors in the German general population reflecting the recent state of research. Beyond that, knowledge on openness of the older population in Germany towards internet-based approaches to improve brain health is currently lacking. Against this background, the present study investigates knowledge on risk- and protective factors for dementia and openness towards eHealth interventions for brain health in the general population of Germany aged 60 years and older.

## Materials and methods

### Recruitment and study population

Computer-assisted telephone surveys were conducted between March and April 2022 by USUMA GmbH, an independent social research institute located in Berlin, Germany. The sample size was set at n = 500 individuals at least 60 years of age. Participants were recruited using a multi-stage random digit dialing procedure, drawing upon the Association of German Market and Social Research Agency’s (ADM) sample base, including registered and non-registered landline telephone numbers from the German resident population.

This study was performed in accordance with the principles of the Declaration of Helsinki in its revised version from 2000. The Ethics Committee of the Medical Faculty of the University of Leipzig approved the study (ref.: 587/21-ek). Interviewers informed participants verbally about the study at the beginning of the telephone survey. Participants then provided oral consent, documented electronically by USUMA GmbH.

To ensure a random selection of households, telephone numbers were drawn proportionally to the German population structure and regionally stratified according to district size. If more than one person aged 60 years and older was living in a household, the person to be interviewed was also randomly selected applying the Kish-Selection-Grid. Up to ten attempts were made to reach selected telephone numbers. All interviewers were trained to conduct surveys on health-related topics and interviewers were randomly monitored for quality control. After training of interviewers, a pretest of the final survey was undertaken to ensure validity, reliability and discriminatory power of survey items, using quota sampling based on participant’s age, sex and geographic region.

Sample size was determined based on comparable studies investigating dementia literacy in older population- or community-based settings, e.g. [9] (n = 590), [28] (n = 385) or [29] (n = 312). As our study did not aim to test explicit hypotheses, a sample size of n = 500 was deemed sufficient. Current evidence suggests that the optimal window of opportunity for primary prevention of dementia is between 60 and 77 years [30,31]. Since we aimed to assess openness towards eHealth interventions for brain health as a measure of primary prevention, we focused on adults aged 60 years and older to determine willingness to use respective interventions in older adults from the general population.

### Weighting procedures

To maximize representativeness of the sample, a weighting factor was applied by USUMA GmbH to adjust for differences from the German general population in terms of age, sex and regions across Germany. Data on population statistics were provided by the German Federal Statistical Office. A design weighting procedure was applied to account for disproportionate probability of selection for people in smaller households. In this study, we present unweighted absolute frequencies, while any further analyses were conducted applying the weighting factor.

### Instruments

#### Sociodemographic information

During each survey, information on participants’ sex, age, relationship status, country of birth, education and occupational status was assessed using a standardized questionnaire.

#### Knowledge and attitudes on dementia and dementia prevention

Surveys were based on a standardized instrument using closed and open-ended questions, assessing the following topics:

- self-perceived knowledge on dementia and belief in preventability
- knowledge of risk and protective factors for dementia
- preferences for information on dementia risk reduction
- openness towards internet-based prevention programs against dementia

At the opening of the survey, participants were asked whether they believed in the preventability of dementia (“Do you think dementia could be prevented?”, response options: yes, no), followed by an open question if respondents answered “yes” (“If yes, what could prevent dementia?”). Respective questions were drawn from a previous study on dementia literacy in Germany [10]. Closed questions assessing knowledge on established risk and protective factors for dementia were derived from a recent population-based survey conducted in the Netherlands [9], supplemented by further established risk factors as described by the Lancet Commission on Dementia Prevention and Care [5]. Participants responded to closed-ended statements (e.g. “Smoking increases your chances of getting dementia”) on a five-point Likert-scale, with the response options “agree strongly”, “agree”, “neither agree nor disagree”, “disagree” and “disagree strongly”. To check for monotone answering tendencies possibly limiting evaluability, two sham questions were included asking about factors without known associations with dementia risk (having children; poor personal hygiene). Preferences for information on brain health were assessed asking “Would you be interested in receiving information on how to improve your brain health?“, with the response options being “yes”, “no” and “maybe”. Openness towards eHealth interventions was assessed asking the question: “In the case that there was a web-based application, that means a website or an app, providing you without charge with information about your brain health and giving advice on how to improve your brain health, would you use this application?”. Participants were informed that the survey assessed general interest in the topic, without the intent to sell a specific product or service. Lastly, the questionnaire assessed preferred sources of professional help regarding dementia (“What would be your first source of professional help?”, response options: general practitioner, neurologist, psychiatrist, specialized services like memory clinics, other). The respective results are presented in a separate publication (results submitted).

#### Other covariates

To control for further factors possibly linked to willingness of using eHealth applications for dementia risk reduction, we assessed health literacy, using the Health Literacy Survey-EU-Q16 (HLS-EU-Q16; [32]). Since dementia might constitute a potentially stressful topic for older adults, we further assessed resilience using the Brief Resilience Scale (BRS; [33]), as resilience has been linked to active coping styles [34], self-efficacy and internal locus of control [35], which might facilitate dealing with burdensome topics. An English translation of the full questionnaire is provided in S1 File.

#### Statistical analyses

We conducted group comparisons using Chi^2^- and t-tests as appropriate. Factors linked to openness towards eHealth interventions for brain health were assessed using multivariable logistic regression. As recent studies suggest a pronounced “digital divide” between younger seniors and older adults aged 75 years and older (for an overview, please see [20]), regression analyses on preferences for information on brain health and openness towards eHealth interventions were conducted stratified by age group (total sample; adults aged ≤ 75 years). Analyses were conducted using Stata 16.0 SE (StataCorp, College Station, TX, USA) and an alpha-level of 0.05 (two-tailed) was chosen to indicate significance.

## Results

### Sample characteristics

Of 1.067 individuals who were initially selected, 244 (22.9%) could not be reached and 320 (30.0%) refused to participate, leading to a sample of n = 503 individuals (response rate: 47.1). Three participants endorsed all 18 risk factors and the two sham-items and were therefore excluded due to suspected monotone answering tendencies. The final sample therefore consisted of n = 500 observations. Older participants had more missing values on the covariates health literacy and resilience (p = .0016 and .026, respectively). Numbers of missing values of covariates did not differ by sex, level of education, employment status or country of birth. Table 1 provides a description of the final analysis sample.

**Table 1:** Description of the study sample (n = 500).

Demographic information	Mean (SD) / n (%)
Age in years	74.8 (9.0)
60–69 years	169 (33.8)
70–79 years	160 (32.0)
80–98 years	171 (34.2)
Female	314 (62.8)
Marital status	
Married/living in a partnership	207 (41.6)
single	77 (15.5)
divorced	58 (11.7)
widowed	156 (31.3)
Education	
low	91 (18.3)
middle	185 (37.2)
high	221 (44.5)
Employment	
retired	426 (85.2)
employed, part-time or full-time	64 (12.8)
unemployed	3 (0.6)
homemaker	7 (1.4)
Country of birth	
Germany	457 (91.6)
other	42 (8.4)
Brief Resilience Scale Score (points)	3.6 (.7)
Health Literacy	
inadequate	35 (7.7)
problematic	126 (27.6)
sufficient	295 (64.7)

Missing values: Marital status– 2 (0.4%); country of birth– 1 (0.2%); education– 3 (0.6%); resilience– 30 (6.0%%); health literacy: 44 (8.8%); Table presents absolute numbers (percentages) or means (SD).

#### Self-perceived knowledge on dementia and belief in preventability

The majority of participants (69.6%) stated that they knew / had known a person with dementia, naming a friend or acquaintance (37.4%), a partner, parent or child (22.8%), a grandparent (6.6%), a colleague (5.0%), or other (5.0%; multiple responses possible). When asked about their knowledge on dementia, 15.4% stated to know “very much”, 35.6% “a lot”, 34.8% “something”, 10.8% “rather little” and 3.0% “nothing” about dementia (“I don’t know”: 0.4%). Two thirds of participants (67.9%) believed that dementia can be prevented (missing values: 11.6%). Belief in preventability was higher among women than among men (72.2% vs. 60.3%, p = .009), but did not differ between levels of education (education low/middle/high: 63.2%/66.3%/71.9%, respectively; p = .291). Older participants did not differ from younger ones regarding belief in preventability of dementia (p = .183).

### Knowledge on risk and protective factors for dementia

Risk and protective factors as endorsed by participants are described in Table 2. Response options were dichotomized, with responses “agree strongly” and “agree” indicating endorsement of the respective risk/protective factor. Data on responses to the open question on preventability of dementia are available upon request.

**Table 2:** Risk and protective factors for dementia named by participants.

Risk/protective factor	% endorsed	n (total = 500)
Physical activity	86.9	490
Cognitive activity	86.7	495
Social isolation	82.0	489
Education and lifelong learning	74.8	492
Healthy diet	70.0	490
Depression	68.8	475
Low alcohol consumption	61.8	482
Having a parent with dementia	58.1	472
Traumatic brain injury	53.8	444
Smoking	53.5	458
Hearing loss	43.3	467
Elevated cholesterol	39.4	429
Diabetes	38.2	422
Hypertension	36.1	438
Obesity	31.2	468
Air pollution	22.8	439
Poor personal hygiene (sham-item)	22.0	477
Heart disease	21.0	433
Chronic kidney disease	16.9	402
Having children (sham-item)	2.3	483

sham-items highlighted in color.

Knowledge on certain risk factors varied by gender. Men more often correctly named diabetes (p < .001), obesity (p = .027), smoking (p = .006), hypertension (p = .001) and a healthy diet (p = .010) as risk or protective factors. Participants with higher levels of education more often named having a parent with dementia (p < .001), elevated cholesterol (p = .046), healthy nutrition (p = .020), hearing loss (p = .041), obesity (p = .018), social isolation (p = .047), physical activity (p < .001) and smoking (p = .009) as risk/protective factors. On the other hand, participants with lower levels of education were more likely to name air pollution as a risk factor (p = .008). Older participants named chronic kidney disease (p = .005) more often than younger participants, but were also more likely to endorse poor personal hygiene (sham-item) as a risk factor for dementia (p = .002). Detailed information on sociodemographic covariates of endorsement of the respective risk and protective factors is provided in **S 2**.

Numbers of missing values were higher in older participants for the risk factors “having a parent with dementia” (p = .042), “diabetes” (p = .001), “heart disease” (p = .001) and “elevated cholesterol” (p = .005). Respondents who were single or widowed more often had missing values for the risk factors “diabetes” (p = .027) and “heart disease” (p = .014), while participants with high levels of education had more missing values for the risk factor “poor personal hygiene” (sham-item; p = .043). No differences by sex, employment status or country of birth were detected for missing values on any of the respective risk/protective factors.

### Preferences for information on dementia risk reduction

Regarding the wish for information on brain health, 23.1% of respondents answered “yes” and 15.0% “maybe” (Table 3). Among these participants (n = 190), 53.2% preferred researching on the internet for more information. Almost half (46.3%) named their GP as preferred source of information. A minority (32.6%) would consider information from professional health organizations, e.g. the German Federal Ministry of Health or the German Alzheimer’s Association. Scientific publications and libraries were endorsed by 38.4% and 29.0% of participants, respectively. Other sources of information named by participants included talking to friends and family, radio and television, books, self-help groups and pharmacies.

**Table 3:** Preferences for information on brain health and preferred sources of information.

Item	N (%; total: 500)
**Wish for more information**	499
Yes	115 (23.1)
Maybe	75 (15.0)
No	309 (61.9)
**Preferred source of information** ^#^	190*
General practitioner	88 (46.3)
Search on the internet	101 (53.2)
Information from professional organizations	62 (32.6)
Scientific publications	73 (38.4)
Library	55 (29.0)
None of the above	9 (4.7)
Other	14 (7.4)

^#^ multiple responses possible;

* proportion of total sample expressing the wish for more information on brain health.

We applied logistic regression analysis to assess factors linked towards a wish for further information on brain health. Results of weighted logistic regression analyses are displayed in Table 4.

**Table 4:** Factors associated with interest in information on brain health.

	Total sample (n = 426)			Age ≤ 75 (n = 240)		
Variable	OR	SE	95% CI	OR	SE	95% CI
Age in years	**1.03**	.01	1.00; 1.06	1.07	.04	.99; 1.16
Female (ref: male)	.79	.19	.49; 1.26	.75	.24	.41; 1.39
Marital status (ref.: married)						
Single	.68	.24	.34; 1.34	.84	.35	.37; 1.92
Divorced	.81	.28	.42; 1.58	.56	.27	.22; 1.42
Widowed	**.35**	.10	.20; .62	**.25**	.12	.10; .66
Education (ref: low)						
Moderate	.55	.18	.29; 1.05	.**37**	.18	.14; .98
High	.67	.21	.36; 1.25	.46	.22	.18; 1.16
Subjective knowledge on dementia (ref: very much)						
A lot	.95	.31	.50; 1.81	1.14	.52	.46; 2.81
Something	1.07	.35	.56; 2.05	1.41	.64	.58; 3.43
Rather little	.65	.30	.26; 1.61	1.08	.73	.28; 4.09
Nothing	.53	.42	.11; 2.54	#		
Health literacy (HLS-EU-Q16; ref: inadequate)						
Problematic	**.34**	.17	.13; .90	.44	.32	.11; 1.83
Sufficient	**.34**	.15	.13; .85	.50	.34	.13; 1.87
Resilience (BRS-Score)	.93	.16	.66; 1.31	.95	.23	.60; 1.51
Knowledge of risk and protective factors (sum score)	**1.11**	.04	1.04; 1.19	**1.11**	.05	1.01; 1.22
Knowing someone with dementia (ref.: no)	1.28	.36	.74; 2.21	1.53	.63	.68; 3.46
Openness towards early diagnosis (ref.: no)	1.02	.02	.98; 1.07	**1.38**	.16	1.10; 1.72

Significant associations in bold type; education assessed according to CASMIN (Comparative Analysis of Social Mobility in Industrial nations); BRS: Brief Resilience Scale; HLS-EU-Q16: Health Literacy Scale EU-Q16; CI: Confidence interval; SE: Standard error; observations with missing values on dependent variable or any independent variable were excluded from analysis.

^#^: No observations with this expression in subsample ≤ 75 years.

Older age was associated with the wish for more information on brain health (OR = 1.03, 95% CI: 1.00; 1.06), as was better knowledge of risk and protective factors for dementia (OR = 1.11; 95% CI: 1.04; 1.19). Being widowed (OR: .35; 95% CI: .20; .62) and having problematic (OR: .34; 95% CI: .13; .90) or sufficient (OR: .34; 95% CI: .13; .85) levels of health literacy was linked to lower interest in information on brain health. In the subsample of participants aged up to 75 years, being widowed (OR: .25; 95% CI: .10; .66) and a moderate level of education (OR: .37; 95% CI: .14; .98) was linked to lower interest in information on brain health. Better knowledge of risk and protective factors (OR: 1.11; 95% CI: 1.01; 1.22) and openness towards an early diagnosis of dementia (OR: 1.38; 95% CI: 1.10; 1.72) were linked to a wish for more information on brain health.

Examined individually, better knowledge of risk and protective factors and openness towards an early diagnosis of dementia were linked to the wish for more information on brain health, while women, widowed respondents and those with sufficient health literacy had lower interest in respective information. Detailed results of univariable regression analyses are provided in **S 2**.

#### Openness towards internet-based prevention programs against dementia

In a next step, we assessed participants’ openness towards eHealth interventions for brain health. Half of participants (53.7%) stated not to be interested in using either an app or a website for information on brain health and ways to reduce risk for dementia. 3.4% were open towards using an app, 16.0% would consider using a website, 11.2 were open towards both options, while further 15.6% answered “maybe” (missing values: n = 1, .2%).

We applied logistic regression analysis to investigate factors associated with openness towards eHealth interventions for brain health (openness dichotomized as “yes” towards either an app, a website, both or “maybe” vs. “no”). Weighted logistic regression results are displayed in Table 5.

**Table 5:** Factors associated with openness towards eHealth interventions for brain health.

	Total sample (n = 426)			Age ≤ 75 (n = 240)		
Variable	OR	SE	95% CI	OR	SE	95% CI
Age in years	**.94**	.02	.91; .97	.99	.04	.91; 1.08
Female (ref: male)	1.29	.36	.75; 2.22	1.67	.58	.76; 3.23
Marital status (ref.: married)						
Single	1.04	.36	.45; 1.99	.80	.37	.32; 1.97
Divorced	1.07	.43	.49; 2.37	.69	.39	.23; 2.09
Widowed	.96	.31	.51; 1.82	**.37**	.18	.14; .98
Education (ref: low)						
Moderate	1.72	.68	.80; 3.71	2.53	1.32	.77; 7.08
High	1.79	.68	.85; 3.78	3.19	1.42	.91; 7.57
Subjective knowledge on dementia (ref: very much)						
A lot	1.41	.53	.67; 2.97	1.57	.80	.58; 4.24
Something	1.93	.76	.89; 4.18	1.78	.93	.63; 4.98
Rather little	1.15	.57	.44; 3.04	2.07	1.44	.53; 8.10
Nothing	1.78	1.37	.39; 8.02	#		
Health literacy (HLS-EU-Q16; ref: inadequate)						
Problematic	1.12	.58	.40 3.10	.59	.39	.16; 2.18
Sufficient	1.10	.54	.42; 2.89	.55	.31	.18; 1.67
Resilience (BRS-Score)	.99	.19	.68; 1.44	1.13	.28	.70; 1.82
Knowledge of risk and protective factors (sum score)	**1.10**	.04	1.02; 1.19	**1.13**	.06	1.02; 1.25
Wish for information on brain health (ref: no)	**11.76**	3.28	6.80; 20.31	**13.33**	5.41	6.02; 29.53
Knowing someone with dementia (ref.: no)	**2.03**	.59	1.15; 3.58	**2.90**	1.20	1.29; 6.54
Openness towards early diagnosis (ref.: no)	.99	.02	.95; 1.02	.98	.02	.95; 1.02

Significant associations in bold type; education assessed according to CASMIN (Comparative Analysis of Social Mobility in Industrial nations); BRS: Brief Resilience Scale; HLS-EU-Q16: Health Literacy Scale EU-Q16; CI: Confidence interval; SE: Standard error; observations with missing values on dependent variable or any independent variable were excluded from analysis.

^#^: No observations with this expression in subsample ≤ 75 years.

In the total sample, openness towards using an eHealth intervention for brain health was linked to younger age (OR = .94; 95% CI: .91, .97) and better knowledge of risk and protective factors for dementia (OR = 1.10; 95% CI: 1.02, 1.19). Knowing a person with dementia (OR: 2.03; 95% CI: 1.15, 3.58) and interest in information on brain health (OR: 11.76; 95% CI: 6.80, 20.31) were further associated with openness towards eHealth interventions. When restricting analysis to participants aged up to 75 years, age was no longer associated with openness towards eHealth approaches. Better knowledge of risk and protective factors (OR: 1.13; 95% CI: 1.02, 1.25), knowing someone with dementia (OR: 2.90; 95% CI: 1.29, 6.54) and the wish for further information on brain health (OR: 13.33; 95% CI: 6.02, 29.53) were linked to openness towards eHealth tools.

Younger age, a high level of education, better knowledge of risk- and protective factors and knowing someone with dementia were linked to openness towards eHealth interventions in univariable regression analyses, while being widowed was individually linked to lower openness (see **S 2**).

## Discussion

In this study, we assessed knowledge of potentially modifiable risk and protective factors for dementia in a population-based survey of 500 older community-dwelling adults in Germany. Further, we investigated older adults’ wish for information on brain health and willingness to use eHealth interventions for dementia risk reduction. Belief in preventability of dementia was higher in our sample than previously reported, however, considerable knowledge gaps for certain risk and protective factors for dementia were detected. This especially applied to cardiovascular risk factors, e.g., hypertension, coronary heart disease, elevated cholesterol or diabetes. Interest in receiving information on brain health was moderate, as was openness towards eHealth interventions for dementia risk reduction.

### Self-perceived knowledge on dementia and belief in preventability

Two thirds (67.9%) of older adults interviewed for this study expressed belief that dementia can be prevented, indicating knowledge about a connection between lifestyle and brain health. Belief in preventability was higher than reported in a recent survey conducted in the Netherlands (44%; [9]) and in a systematic review on dementia literacy across several countries (median: 48%; [6], but comparable to recent findings from a Dutch urban population by Vrijsen et al. (62.3%; [8]). The belief that dementia can be prevented was independent of age and education, however, women were more likely to believe that risk for dementia is modifiable. The lack of observed age differences might likely be caused by differences in age ranges between our study and previous investigations; while our study exclusively targeted adults aged 60 and older, other studies reporting age differences in dementia literacy mostly included samples spanning a wider age range [6,7,10].

### Knowledge on risk and protective factors for dementia

In accordance with findings from the Scottish Social Attitudes Survey [36], knowledge of certain risk factors for dementia was slightly better in men. Gender differences were found especially for knowledge of cardiovascular risk factors, e.g., diabetes, hypertension and obesity. These differences could not be explained by differences in level of education between men and women, except for the risk factor obesity (results available upon request). It might be that men had received more information about certain lifestyle risk factors from their attending GPs or other health professionals. A recent population-based study from Germany found that men were more often consulted on nutrition, weight reduction, smoking and alcohol consumption than women [37]. Moreover, research has identified gender differences in the diagnosis and treatment of coronary heart disease, with cardiovascular risk factors for disease noted more often in men [38] and less thorough examinations in women [39]. Our findings indicate the need for more information on cardiovascular risk factors for dementia, particularly among older women. Further, increased attention on cardiovascular conditions in women and respective education of female patients is warranted on the side of GPs, as women are more often affected by dementia.

Highly educated participants more often named certain risk and protective factors linked to individual behavior, e.g. healthy nutrition, smoking, or physical activity, while air pollution was endorsed as a risk factor more often by those with low levels of education. These findings could point towards differences in attribution style or locus of control, with lower-educated individuals relying more strongly on external factors for disease and a lower belief in modifiability of risk factors, as reported in former studies on locus of control and health-beliefs in older adults [40,41].

Regarding dementia literacy, certain established risk and protective factors were less known than others. The vast majority of participants named cognitive and physical activity as protective factors against dementia, which was comparable to recent findings from a population-based study from New Zealand [42]. However, only a minority was aware of the link between cardiovascular diseases and risk factors, e.g., coronary heart disease (21.0%), diabetes (38.2%), elevated cholesterol (39.4%), hypertension (36.1%) and obesity (31.2%), and dementia risk. Our findings are in strong accordance with a recent review, reporting inadequate public knowledge of cardiovascular risk factors for dementia [43]. This finding suggests that the notion “what is good for the heart is good for the brain” is not yet common among the older general population in Germany. Knowledge of detrimental or protective effects of smoking, low alcohol consumption and a healthy diet for brain health was moderate, as was knowledge of genetic predispositions, i.e., having a parent with dementia, and education. These findings are highly in line with a previous survey where cardiovascular risk factors were least known, while a majority of participants endorsed a physically and cognitively active lifestyle as protective against dementia [9]. Overall, diabetes, depression, a healthy diet, physical activity and low alcohol consumption were correctly identified more often in our study than in the survey of Heger et al. More than 60% of participants in our study correctly stated that depression is linked to increased dementia risk, and more than 80% agreed that social isolation increases the chances of getting dementia. About 20% and 40% identified hearing loss and exposure to air pollution as risk factors, naming two factors only recently established [5]. On the other hand, one fifth, especially among older participants, incorrectly assumed a link between poor personal hygiene and dementia risk. As the WHO dementia risk reduction guidelines emphasize the role of public health campaigns to reduce risk for dementia [44], the observed lack of knowledge on cardiovascular risk factors for dementia indicate a need for more targeted information for older adults to increase prevention potential. Raising awareness about the link between the respective conditions and brain health might be a promising strategy to motivate older adults to endorse healthy lifestyle changes also beneficial for brain health.

### Preferences for information on dementia risk reduction

While the majority of participants believed in the preventability of dementia, this did not necessarily imply a wish for more information. Only a minority (38.5%) wanted to know more about individual dementia risk and possible preventive strategies, which was lower than in the study by [9]. Strikingly, those who identified more risk and protective factors were also more likely to be open to further information on dementia risk reduction. Since awareness and risk perception increase willingness to change behavior [13,45], this indicates a need for targeted information on risk and protective factors for dementia. Beyond that, however, more information on individual-level factors, e.g., older adults’ attitudes and self-efficacy expectations when considering brain-healthy lifestyles, is needed to design and implement effective intervention campaigns which empower people to actively take charge of their brain health. Interestingly, however, higher levels of health literacy (i.e. problematic or sufficient compared to inadequate) were linked to lower interest in dementia risk reduction. These findings point towards an interest in information on dementia risk reduction tailored specifically to older adults with low health literacy. Resilience was not linked to interest in information on brain health, indicating that the wish to be informed about dementia risk reduction was independent of the ability to deal with stress in our sample. Future studies investigating, e.g., personality type or coping style on information preferences might provide further insights on which personal characteristics influence older peoples’ information preferences regarding brain-healthy ageing. Further, being widowed was linked to lower openness towards information on dementia risk reduction. This might be due to differing levels of perceived social support or motivation for behavior change depending on relationship status, making those living alone less interested in information on dementia risk reduction. However, as we did not directly assess motivation to change behavior, this line of thought should be interpreted with caution. Restricting the sample to older adults ≤ 75 years of age, openness towards an early diagnosis for dementia was linked to greater interest in information on brain health.

On the other hand, it is important to consider potential negative consequences such as fear and distress on the side of the individual when being informed about dementia and acknowledge different preferences and attitudes of older adults regarding knowledge of the condition [46]. Future studies are warranted to further investigate the factors that determine older people’s wish to learn about dementia and possible ways to reduce individual risk for disease in order to facilitate the design of effective prevention campaigns, which take into account different needs and preferences of older adults.

### Openness towards internet-based prevention programs against dementia

Only a minority of participants expressed openness towards eHealth interventions, which was lower than reported in a previous study from the Netherlands [9]. These differences might partly be explained by higher levels of interest in information on dementia risk reduction in the sample of Heger et al., possibly leading to greater openness towards eHealth approaches. Further, the mean age in our sample was rather high (74.8 years). Although usage of computers, mobile devices and the internet in general is increasing rapidly among older age groups in Germany [20,47], eHealth tools specifically targeting older adults still constitute a rather recent approach. The questions in our survey assessed general interest in a potential eHealth tool without offering further illustrative material, possibly influencing participants’ responses. When restricting the sample to older adults aged 60 to 75 years, openness towards eHealth tools increased slightly. Knowledge on risk and protective factors for dementia, knowing or having known someone with dementia and interest in more information on brain health was linked to greater openness towards eHealth intervention. These findings are in line with a recent study by Akyol et al., reporting that a family history of dementia increased readiness to change behavior in order to reduce dementia risk [48]. Neither health literacy nor resilience were associated with openness towards eHealth interventions. Including factors like, e.g., internet competence or motivation to change behavior might be suitable covariates to be included in future studies on eHealth interventions for older adults.

Our results suggests that the idea of internet-based approaches for health in older age currently appeals to a minority of older adults already interested in the topic, indicating the risk of a “digital divide” previously described for internet use in older generations [49,50]. Choosing a different sampling approach, e.g., by targeting older adults who regularly interact with computers and mobile devices, might have revealed different results. A qualitative study investigating experts’ views on possible facilitators and barriers of eHealth use for brain health is currently conducted by the authors and will provide valuable information on the potential of respective approaches in the target group of older adults.

### Strengths and limitations

To the best of our knowledge, our study is the first to describe knowledge on modifiable risk and protective factors for dementia in the German general population, assessing the current state of research. We used data from a population-based sample of older adults living in Germany, which should make our results more robust against selection bias, a problem often encountered in studies using convenience sampling. We assessed knowledge of specific, well-established risk factors for dementia rather than using open questions on preventability, thereby providing evidence for the design of targeted information and prevention efforts for future public health approaches. Lastly, our study is the first to provide information on openness of older adults in Germany towards an innovative approach against cognitive decline and dementia, i.e., eHealth interventions.

Certain limitations need to be addressed when interpreting our findings. Mention of the study topic during the introduction of the survey might have deterred certain older adults. It cannot be ruled out that older people who are especially concerned about dementia or who think that the disease is inevitable might have been less likely to participate, possibly introducing selection bias. Reasons for refusal of participation were not recorded, therefore we cannot rule out possible differences between responders and non-responders. Although we used a population-based sample, derived using a multi-stage random sampling procedure, average level of education was rather high in our sample (% high education: 44.5), which might have introduced selection bias and may have influenced our findings. Including higher proportions of older adults with low levels of education may have resulted in lower identification rates of risk and protective factors for dementia, therefore, our findings might provide rather optimistic estimates. Older adults were contacted via landline telephone numbers, excluding mobile telephone numbers. This may have resulted in a selected sample, as older adults using (solely) mobile phones who might be more internet- and computer-literate were less likely to be included in our study sample. However, since coverage of households with older adults by landline numbers is very high in Germany, we are confident that the potential bias introduced by our sampling strategy did not substantially influence results.

Further, we did not assess usage of computers or smartphones among participants, or collect information on internet access. Recent evidence from the HATICE-trial found usage of computers in the months previous to the trial to predict engagement with and adherence to the eHealth-intervention [51]. It is likely that computer skills and access to the internet might have influenced participants’ openness towards an eHealth intervention targeting brain health. Future studies should control for participants’ familiarity with electronic devices and the internet to identify target groups for eHealth interventions and to adapt respective approaches towards the needs and prerequisites of older adults.

## Conclusion

Our study revealed knowledge gaps in highly prevalent risk factors for dementia, especially cardiovascular conditions, in older adults living in Germany. Improving knowledge on these modifiable lifestyle factors and their link to dementia risk might help promote preventative health behaviors such as physical activity and management of diabetes and hypertension. Pointing out the benefits of a healthy lifestyle not only for the respective cardiovascular diseases but also for brain health might provide an additional motivation for older adults. However, not all older adults express the wish to learn about dementia risk reduction. Further research is needed on how to best disseminate scientific evidence to the wider public, but also to assess why certain groups express skepticism towards preventive approaches against dementia and how to handle the different needs of older adults regarding dementia and disease prevention. Although openness towards eHealth applications for brain health was only moderate in our sample, respective tools constitute a promising approach for cost-effective management of dementia risk factors. As internet use in those 60 years and older is increasing rapidly, further investigations identifying the needs and wishes of older adults in this regard are highly warranted to aid in the design and implementation of innovative intervention approaches tailored to the needs of older adults.

## Supporting information

S1 File English translation of the full questionnaire. (DOCX)

S2 File Supplementary analyses. (DOCX)
