# Supplementary material for: Knowledge of risk and protective factors for dementia in older German adults A population-based survey on risk and protective factors for dementia and internet-based brain health interventions
Source: PLoS One. 2022 Nov 7;17(11):e0277037. doi: 10.1371/journal.pone.0277037 (PMC9639821; doi:10.1371/journal.pone.0277037)
Supplement: S1 File — (DOCX) [file pone.0277037.s001.docx]

**Appendix 1: English translation of the survey questionnaire**

| **1) First, I would ask you to tell me your gender.** | |
| --- | --- |
| Male | □ |
| Female | □ |
| Other, please specify: | □ |

| **2) Please tell me your age.** | |
| --- | --- |
| Age in years |  |

| **3) Please tell me your current marital status.** | |
| --- | --- |
| Married, cohabitating 🡪 continue with question 5 | □ |
| Married, living apart | □ |
| Single | □ |
| Divorced | □ |
| widowed | □ |

| **4) Do you live in a steady partnership?** | |
| --- | --- |
| Yes | □ |
| No | □ |

| **5) Now we will begin with the actual topic of the interview. First, I would like to know how you personally rate your knowledge on dementia.** | |
| --- | --- |
| **Dementia comprises several symptoms such as loss of memory, mood swings, not remembering names or trouble finding words. Alzheimer’s disease is one form of dementia. How would you rate your knowledge on dementia – would you say you know…** | |
| Very much | □ |
| A lot | □ |
| Something | □ |
| Rather little | □ |
| Nothing | □ |
| **…about dementia?** |  |
| I don’t know | □ |
| I prefer not to answer that question | □ |

| **6) Personally, do you know somebody who has or had dementia? Please indicate all options that apply.** | |
| --- | --- |
| No, I don’t know anybody who has/had dementia | □ |
| Yes, my partner, a parent or a child | □ |
| Yes, a grandparent | □ |
| Yes, a friend or acquaintance | □ |
| Yes, a colleague/somebody at work | □ |
| Yes, someone else, please specify: | □ |
| I don’t know | □ |
| I prefer not to answer that question | □ |

| **7) Do you think that early detection of dementia should be offered?** | |
| --- | --- |
| Yes | □ |
| No | □ |

| **8) Would you be willing to be examined for early diagnosis of dementia?** | |
| --- | --- |
| No | □ |
| Less likely | □ |
| Undecided | □ |
| More likely | □ |
| In all cases | □ |

| **9) Do you think that dementia could be prevented? If yes, what could prevent dementia?** | |
| --- | --- |
| Yes, please specify: | □ |
| No | □ |

| **10) What would be your first source of professional help?** | |
| --- | --- |
| General practitioner | □ |
| Neurologist | □ |
| Psychiatrist | □ |
| Specialized services like memory clinics | □ |
| Other, please specify: | □ |
| I don’t know | □ |

I will now read to you several statements. For each statement, please indicate to what extent you agree – do you agree fully, or do you disagree fully? You can grade your answer with the options inbetween.

| **11) There is nothing anyone can do to reduce their risks of getting dementia.** | |
| --- | --- |
| Agree strongly | □ |
| Agree | □ |
| Neither agree nor disagree | □ |
| Disagree | □ |
| Disagree strongly | □ |

| **12) High blood pressure increases your chances of getting dementia.** | |
| --- | --- |
| Agree strongly | □ |
| Agree | □ |
| Neither agree nor disagree | □ |
| Disagree | □ |
| Disagree strongly | □ |

| **13) Having a parent with dementia increases your chances of getting dementia.** | |
| --- | --- |
| Agree strongly | □ |
| Agree | □ |
| Neither agree nor disagree | □ |
| Disagree | □ |
| Disagree strongly | □ |

| **14) Head injuries increase your chances of getting dementia.** | |
| --- | --- |
| Agree strongly | □ |
| Agree | □ |
| Neither agree nor disagree | □ |
| Disagree | □ |
| Disagree strongly | □ |

| **15) Smoking increases your chances of getting dementia.** | |
| --- | --- |
| Agree strongly | □ |
| Agree | □ |
| Neither agree nor disagree | □ |
| Disagree | □ |
| Disagree strongly | □ |

| **16) No or moderate alcohol use lowers your chances of getting dementia.** | |
| --- | --- |
| Agree strongly | □ |
| Agree | □ |
| Neither agree nor disagree | □ |
| Disagree | □ |
| Disagree strongly | □ |

| **17) Regular physical activity lowers your chances of getting dementia.** | |
| --- | --- |
| Agree strongly | □ |
| Agree | □ |
| Neither agree nor disagree | □ |
| Disagree | □ |
| Disagree strongly | □ |

| **18) Social isolation increases your chances of getting dementia.** | |
| --- | --- |
| Agree strongly | □ |
| Agree | □ |
| Neither agree nor disagree | □ |
| Disagree | □ |
| Disagree strongly | □ |

| **19) Depression increases your chances of getting dementia.** | |
| --- | --- |
| Agree strongly | □ |
| Agree | □ |
| Neither agree nor disagree | □ |
| Disagree | □ |
| Disagree strongly | □ |

| **20) Exposure to air pollution increases your chances of getting dementia.** | |
| --- | --- |
| Agree strongly | □ |
| Agree | □ |
| Neither agree nor disagree | □ |
| Disagree | □ |
| Disagree strongly | □ |

| **21) Diabetes increases your chances of getting dementia.** | |
| --- | --- |
| Agree strongly | □ |
| Agree | □ |
| Neither agree nor disagree | □ |
| Disagree | □ |
| Disagree strongly | □ |

| **22) Being overweight increases your chances of getting dementia.** | |
| --- | --- |
| Agree strongly | □ |
| Agree | □ |
| Neither agree nor disagree | □ |
| Disagree | □ |
| Disagree strongly | □ |

| **23) Poor personal hygiene increases your chances of getting dementia.** | |
| --- | --- |
| Agree strongly | □ |
| Agree | □ |
| Neither agree nor disagree | □ |
| Disagree | □ |
| Disagree strongly | □ |

| **24) A mentally active lifestyle lowers the chances of getting dementia.** | |
| --- | --- |
| Agree strongly | □ |
| Agree | □ |
| Neither agree nor disagree | □ |
| Disagree | □ |
| Disagree strongly | □ |

| **25) Hearing loss increases your chances of getting dementia.** | |
| --- | --- |
| Agree strongly | □ |
| Agree | □ |
| Neither agree nor disagree | □ |
| Disagree | □ |
| Disagree strongly | □ |

| **26) Heart disease increases your chances of getting dementia.** | |
| --- | --- |
| Agree strongly | □ |
| Agree | □ |
| Neither agree nor disagree | □ |
| Disagree | □ |
| Disagree strongly | □ |

| **27) Kidney disease increases your chances of getting dementia.** | |
| --- | --- |
| Agree strongly | □ |
| Agree | □ |
| Neither agree nor disagree | □ |
| Disagree | □ |
| Disagree strongly | □ |

| **28) Having children increases your chances of getting dementia.** | |
| --- | --- |
| Agree strongly | □ |
| Agree | □ |
| Neither agree nor disagree | □ |
| Disagree | □ |
| Disagree strongly | □ |

| **29) Education and lifelong learning lowers the chances of getting dementia.** | |
| --- | --- |
| Agree strongly | □ |
| Agree | □ |
| Neither agree nor disagree | □ |
| Disagree | □ |
| Disagree strongly | □ |

| **30) High cholesterol increases your chances of getting dementia.** | |
| --- | --- |
| Agree strongly | □ |
| Agree | □ |
| Neither agree nor disagree | □ |
| Disagree | □ |
| Disagree strongly | □ |

| **31) A healthy diet lowers the chances of getting dementia.** | |
| --- | --- |
| Agree strongly | □ |
| Agree | □ |
| Neither agree nor disagree | □ |
| Disagree | □ |
| Disagree strongly | □ |

| **32) Next, we would like to know how interesting certain topics are to you. We do not want to offer or sell you a specific product or service. Would you be interested in receiving information on how to improve your brain health?** | |
| --- | --- |
| Yes | □ |
| No | □ |
| Maybe | □ |

| **33) In the case that you would want to know more about your own brain health, what information channels would you like to use to find this information? Please choose all that apply.** | |
| --- | --- |
| Search on the web | □ |
| GP practice | □ |
| Information provided by specialist organisations (e.g. German Federal Ministry of Health, Deutsche Alzheimergesellschaft) | □ |
| Scientific publications | □ |
| Library | □ |
| None of the above | □ |
| Somewhere else, please specify: | □ |
| I don’t know | □ |
| I prefer not to answer that question | □ |

| **34) In the case that there was a web-based application, that means a website or an app, providing you without charge with information about your brain health and giving advice on how to improve your brain health, would you use this application?** | |
| --- | --- |
| Yes, I would use both | □ |
| Yes, I would use an app | □ |
| Yes, I would use a website | □ |
| No, I would use neither | □ |
| Maybe | □ |
| I prefer not to answer that question | □ |

| **35) In the following questions, we would like to know how you usually deal with stressful situations. How strongly do you agree or disagree with the following statements:** | | | | | |
| --- | --- | --- | --- | --- | --- |
|  | **Strongly disagree** | **Disagree** | **Neutral** | **Agree** | **Strongly agree** |
| I tend to bounce back quickly after hard times. | □ | □ | □ | □ | □ |
| I have a hard time making it through stressful events. | □ | □ | □ | □ | □ |
| It does not take me long to recover from a stressful event. | □ | □ | □ | □ | □ |
| It is hard for me to snap back when something bad happens. | □ | □ | □ | □ | □ |
| I usually come through difficult times with little trouble. | □ | □ | □ | □ | □ |
| I tend to take a long time to get over setbacks in my life. | □ | □ | □ | □ | □ |

| **36) The next questions refer to health-related activities and behaviors which can be difficult. For each behavior, please indicate how easy or difficult you find to perform it. On a scale from very easy to very difficult, how easy would you say it is to…** | | | | |
| --- | --- | --- | --- | --- |
|  | **Very easy** | **Fairly easy** | **Fairly difficult** | **Very difficult** |
| …find information on treatments of illnesses that concern you? | □ | □ | □ | □ |
| …find out where to get professional help when you are ill? | □ | □ | □ | □ |
| …understand what your doctor says to you? | □ | □ | □ | □ |
| . …understand your doctor’s or pharmacist’s instruction on how to take a prescribed medicine? | □ | □ | □ | □ |
| …judge when you may need to get a second opinion from another doctor? | □ | □ | □ | □ |
| …use information the doctor gives you to make decisions about your illness? | □ | □ | □ | □ |
| …follow instructions from your doctor or pharmacist? | □ | □ | □ | □ |
| …find information on how to manage mental health problems like stress or depression? | □ | □ | □ | □ |
| …understand health warnings about behaviour such as smoking, low physical activity and drinking too much? | □ | □ | □ | □ |
| …understand why you need health screenings? | □ | □ | □ | □ |
| …judge if the information on health risks in the media is reliable? | □ | □ | □ | □ |
| …decide how you can protect yourself from illness based on information in the media? | □ | □ | □ | □ |
| …find out about activities that are good for your mental well-being? | □ | □ | □ | □ |
| …understand advice on health from family members or friends? | □ | □ | □ | □ |
| …understand information in the media on how to get healthier? | □ | □ | □ | □ |
| …judge which everyday behaviour is related to your health? | □ | □ | □ | □ |

At the end of the interview, we would like to ask you some additional questions on your personal and professional background.

| **37) Please tell me your height.** | |
| --- | --- |
| Height in centimeters | ___ |

| **38) Would you tell me your current weight?** | |
| --- | --- |
| Bodyweight in kilogram | __ |

| **40) Which is your highes degree of formal education?** | |
| --- | --- |
| Do you have… | □ |
| A high school diploma (Hauptschule / Volksschule)? | □ |
| A secondary school certificate (Mittlere Reife)? | □ |
| A polytechnic secondary school certificate? | □ |
| An advanced technical college certificate? | □ |
| A general or subject-specific qualification for university entrance? | □ |
| Other degree, please specify**:**  ___________________________ | □ |

| **41) Which is your highes degree of professional education?** | |
| --- | --- |
| Do you have… |  |
| No professional qualification and are not currently in professional training? |  |
| Vocational training? | □ |
| School-based professional training? | □ |
| A degree from a vocational school, technical college or professional academy? | □ |
| A university or polytechnic degree | □ |
| Another degree, please specify: | □ |

| 42) Are you currently employed? | |
| --- | --- |
| Yes 🡪 continue with question 43 | □ |
| No (including unemployment or professional training) 🡪 continue with question 44 | □ |

| **43) Are you…** | |
| --- | --- |
| A low or mid-level employee / official? | □ |
| A senior executive / higher official? | □ |
| An unskilled laborer? | □ |
| A skilled worker, foreman? | □ |
| Self-employed? | □ |
| An unpaid family worker? | □ |
| Something else, please specify: | □ |

| **44) Are you…** | |
| --- | --- |
| Unemployed? | □ |
| A homemaker? | □ |
| Retired? | □ |
| Something else, please specify: | □ |

| **45) Were you born in Germany? This implies the current territory of the Federal Republic of Germany, including the former GDR.** | |
| --- | --- |
| Yes | □ |
| No | □ |

Thank you very much for participating in our survey!
