# Supplementary material for: Knowledge of risk and protective factors for dementia in older German adults A population-based survey on risk and protective factors for dementia and internet-based brain health interventions
Source: PLoS One. 2022 Nov 7;17(11):e0277037. doi: 10.1371/journal.pone.0277037 (PMC9639821; doi:10.1371/journal.pone.0277037)
Supplement: S2 File — (DOCX) [file pone.0277037.s002.docx]

**Appendix 2**

**Supplementary Table 1: Sociodemographic characteristics and endorsement of risk / protective factors for dementia**

| **Risk / protective factor** | **Endorsed (%; total: n = 426)** | **Not endorsed (%; total: n = 64)** |
| --- | --- | --- |
| **Physical activity** |  |  |
| Age in years |  |  |
| 60-69 years | 34.5 | 32.8 |
| 70-79 years | 32.2 | 31.3 |
| 80-98 years | 33.3 | 35.9 |
| Women | 61.5 | 71.9 |
| Men | 38.5 | 28.1 |
| Marital status |  |  |
| married | 42.5 | 37.5 |
| single | 15.6 | 15.6 |
| divorced | 11.6 | 10.9 |
| widowed | 30.4 | 35.9 |
| Education |  |  |
| low | 18.2 | 21.9 |
| middle | 33.6 | 57.8 |
| high | 48.2 | 20.3 |

| **Risk / protective factor** | **Endorsed (%; total: n = 429)** | **Not endorsed (%; total: n = 66)** |
| --- | --- | --- |
| **Cognitive activity** |  |  |
| Age in years |  |  |
| 60-69 years | 35.7 | 21.2 |
| 70-79 years | 30.8 | 40.9 |
| 80-98 years | 33.6 | 37.9 |
| Women | 61.8 | 69.7 |
| Men | 38.2 | 30.3 |
| Marital status |  |  |
| married | 43.7 | 29.2 |
| single | 15.0 | 16.9 |
| divorced | 11.5 | 13.9 |
| widowed | 29.9 | 40.0 |
| Education |  |  |
| low | 17.4 | 25.8 |
| middle | 37.8 | 33.3 |
| high | 44.8 | 40.9 |

| **Risk / protective factor** | **Endorsed (%; total: n = 401)** | **Not endorsed (%; total: n = 88)** |
| --- | --- | --- |
| **Social isolation** |  |  |
| Age in years |  |  |
| 60-69 years | 34.7 | 30.7 |
| 70-79 years | 31.9 | 33.0 |
| 80-98 years | 33.4 | 36.4 |
| Women | 62.6 | 65.9 |
| Men | 37.4 | 34.1 |
| Marital status |  |  |
| married | 44.3 | 29.9 |
| single | 13.8 | 20.7 |
| divorced | 12.5 | 9.2 |
| widowed | 29.5 | 40.2 |
| Education |  |  |
| low | 17.8 | 20.7 |
| middle | 35.8 | 47.1 |
| high | 46.5 | 32.2 |

| **Risk / protective factor** | **Endorsed (%; total: n = 368)** | **Not endorsed (%; total: n = 124)** |
| --- | --- | --- |
| **Education and lifelong learning** |  |  |
| Age in years |  |  |
| 60-69 years | 34.8 | 31.5 |
| 70-79 years | 32.3 | 29.8 |
| 80-98 years | 32.9 | 38.7 |
| Women | 61.7 | 66.9 |
| Men | 38.2 | 33.1 |
| Marital status |  |  |
| married | 43.2 | 38.7 |
| single | 16.1 | 12.9 |
| divorced | 11.5 | 12.1 |
| widowed | 29.3 | 36.3 |
| Education |  |  |
| low | 18.6 | 17.7 |
| middle | 34.3 | 46.0 |
| high | 47.1 | 36.3 |

| **Risk / protective factor** | **Endorsed (%; total: n = 343)** | **Not endorsed (%; total: n = 147)** |
| --- | --- | --- |
| **Healthy diet** |  |  |
| Age in years |  |  |
| 60-69 years | 35.0 | 31.3 |
| 70-79 years | 30.3 | 37.4 |
| 80-98 years | 34.7 | 31.3 |
| Women | 59.2 | 71.4 |
| Men | 40.8 | 28.6 |
| Marital status |  |  |
| married | 41.1 | 42.9 |
| single | 15.0 | 15.7 |
| divorced | 11.4 | 12.2 |
| widowed | 32.6 | 29.3 |
| Education |  |  |
| low | 17.9 | 19.7 |
| middle | 34.1 | 45.6 |
| high | 47.9 | 34.7 |

| **Risk / protective factor** | **Endorsed (%; total: n = 327)** | **Not endorsed (%; total: n = 148)** |
| --- | --- | --- |
| **Depression** |  |  |
| Age in years |  |  |
| 60-69 years | 30.9 | 42.6 |
| 70-79 years | 33.0 | 29.1 |
| 80-98 years | 36.1 | 28.4 |
| Women | 62.1 | 63.5 |
| Men | 37.9 | 36.5 |
| Marital status |  |  |
| married | 42.5 | 41.5 |
| single | 13.8 | 17.0 |
| divorced | 11.6 | 12.2 |
| widowed | 32.1 | 29.3 |
| Education |  |  |
| low | 16.7 | 19.6 |
| middle | 35.2 | 43.9 |
| high | 48.2 | 36.5 |

| **Risk / protective factor** | **Endorsed (%; total: n = 298)** | **Not endorsed (%; total: n = 184)** |
| --- | --- | --- |
| **Low alcohol consumption** |  |  |
| Age in years |  |  |
| 60-69 years | 34.6 | 32.6 |
| 70-79 years | 33.2 | 30.4 |
| 80-98 years | 32.2 | 37.0 |
| Women | 61.1 | 65.8 |
| Men | 38.9 | 34.2 |
| Marital status |  |  |
| married | 41.9 | 41.9 |
| single | 16.9 | 12.5 |
| divorced | 10.8 | 13.0 |
| widowed | 30.4 | 32.6 |
| Education |  |  |
| low | 16.6 | 21.3 |
| middle | 35.1 | 39.9 |
| high | 48.3 | 38.8 |

| **Risk / protective factor** | **Endorsed (%; total: n = 274)** | **Not endorsed (%; total: n = 198)** |
| --- | --- | --- |
| **Having a parent with dementia** |  |  |
| Age in years |  |  |
| 60-69 years | 37.6 | 31.8 |
| 70-79 years | 32.1 | 30.3 |
| 80-98 years | 30.3 | 37.9 |
| Women | 63.1 | 61.1 |
| Men | 36.9 | 38.9 |
| Marital status |  |  |
| married | 41.9 | 39.9 |
| single | 16.2 | 15.2 |
| divorced | 12.1 | 11.1 |
| widowed | 29.8 | 33.8 |
| Education |  |  |
| low | 15.0 | 24.5 |
| middle | 32.6 | 43.4 |
| high | 52.4 | 32.1 |

| **Risk / protective factor** | **Endorsed (%; total: n = 239)** | **Not endorsed (%; total: n = 205)** |
| --- | --- | --- |
| **Traumatic brain injury** |  |  |
| Age in years |  |  |
| 60-69 years | 36.0 | 34.2 |
| 70-79 years | 29.7 | 33.2 |
| 80-98 years | 34.3 | 32.7 |
| Women | 64.0 | 58.5 |
| Men | 36.0 | 41.5 |
| Marital status |  |  |
| married | 40.3 | 44.4 |
| single | 13.9 | 15.1 |
| divorced | 13.0 | 10.7 |
| widowed | 32.8 | 29.8 |
| Education |  |  |
| low | 20.2 | 15.8 |
| middle | 33.2 | 43.4 |
| high | 46.6 | 40.9 |

| **Risk / protective factor** | **Endorsed (%; total: n = 245)** | **Not endorsed (%; total: n = 213)** |
| --- | --- | --- |
| **Smoking** |  |  |
| Age in years |  |  |
| 60-69 years | 31.8 | 38.0 |
| 70-79 years | 33.9 | 30.1 |
| 80-98 years | 34.3 | 31.9 |
| Women | 57.1 | 69.5 |
| Men | 42.9 | 30.5 |
| Marital status |  |  |
| married | 43.0 | 39.6 |
| single | 12.7 | 18.9 |
| divorced | 12.3 | 10.9 |
| widowed | 32.0 | 30.7 |
| Education |  |  |
| low | 16.9 | 18.9 |
| middle | 32.9 | 44.8 |
| high | 50.2 | 36.3 |

| **Risk / protective factor** | **Endorsed (%; total: n = 202)** | **Not endorsed (%; total: n = 265)** |
| --- | --- | --- |
| **Hearing loss** |  |  |
| Age in years |  |  |
| 60-69 years | 29.7 | 37.0 |
| 70-79 years | 32.2 | 31.3 |
| 80-98 years | 38.1 | 31.7 |
| Women | 66.8 | 60.4 |
| Men | 33.2 | 39.6 |
| Marital status |  |  |
| married | 39.5 | 43.8 |
| single | 11.5 | 18.1 |
| divorced | 14.5 | 9.8 |
| widowed | 34.5 | 28.3 |
| Education |  |  |
| low | 19.4 | 18.3 |
| middle | 31.8 | 43.0 |
| high | 48.8 | 38.8 |

| **Risk / protective factor** | **Endorsed (%; total: n = 169)** | **Not endorsed (%; total: n = 260)** |
| --- | --- | --- |
| **Elevated cholesterol** |  |  |
| Age in years |  |  |
| 60-69 years | 33.7 | 36.5 |
| 70-79 years | 28.4 | 34.6 |
| 80-98 years | 37.9 | 28.9 |
| Women | 56.8 | 64.6 |
| Men | 43.2 | 35.4 |
| Marital status |  |  |
| married | 40.7 | 43.9 |
| single | 11.4 | 17.3 |
| divorced | 14.4 | 11.5 |
| widowed | 33.5 | 27.3 |
| Education |  |  |
| low | 15.5 | 20.9 |
| middle | 33.3 | 39.9 |
| high | 51.2 | 39.2 |

| **Risk / protective factor** | **Endorsed (%; total: n = 161)** | **Not endorsed (%; total: n = 261)** |
| --- | --- | --- |
| **Diabetes** |  |  |
| Age in years |  |  |
| 60-69 years | 41.6 | 32.2 |
| 70-79 years | 26.1 | 36.0 |
| 80-98 years | 32.3 | 31.8 |
| Women | 51.6 | 68.6 |
| Men | 48.5 | 31.4 |
| Marital status |  |  |
| married | 50.0 | 39.6 |
| single | 14.4 | 15.4 |
| divorced | 12.5 | 10.8 |
| widowed | 23.1 | 34.2 |
| Education |  |  |
| low | 16.4 | 20.8 |
| middle | 34.0 | 40.4 |
| high | 49.7 | 38.9 |

| **Risk / protective factor** | **Endorsed (%; total: n = 158)** | **Not endorsed (%; total: n = 280)** |
| --- | --- | --- |
| **Hypertension** |  |  |
| Age in years | 31.7 | 36.8 |
| 60-69 years | 32.3 | 30.7 |
| 70-79 years | 36.1 | 32.5 |
| 80-98 years |  |  |
| Women | 52.5 | 68.6 |
| Men | 47.5 | 31.4 |
| Marital status |  |  |
| married | 45.2 | 42.3 |
| single | 16.6 | 14.3 |
| divorced | 13.4 | 10.8 |
| widowed | 24.8 | 32.6 |
| Education |  |  |
| low | 15.9 | 19.4 |
| middle | 34.4 | 38.9 |
| high | 49.7 | 41.7 |

| **Risk / protective factor** | **Endorsed (%; total: n = 146)** | **Not endorsed (%; total: n = 322)** |
| --- | --- | --- |
| **Obesity** |  |  |
| Age in years |  |  |
| 60-69 years | 32.9 | 35.4 |
| 70-79 years | 30.8 | 32.3 |
| 80-98 years | 36.3 | 32.3 |
| Women | 45.2 | 65.5 |
| Men | 54.8 | 34.5 |
| Marital status |  |  |
| married | 40.0 | 43.0 |
| single | 17.2 | 14.3 |
| divorced | 11.0 | 11.8 |
| widowed | 31.7 | 30.8 |
| Education |  |  |
| low | 15.2 | 20.0 |
| middle | 31.7 | 40.9 |
| high | 53.1 | 39.1 |

| **Risk / protective factor** | **Endorsed (%; total: n = 100)** | **Not endorsed (%; total: n = 339)** |
| --- | --- | --- |
| **Air pollution** |  |  |
| Age in years |  |  |
| 60-69 years | 31.0 | 35.4 |
| 70-79 years | 31.0 | 32.2 |
| 80-98 years | 38.0 | 32.5 |
| Women | 55.0 | 64.0 |
| Men | 45.0 | 36.0 |
| Marital status |  |  |
| married | 34.7 | 45.4 |
| single | 16.3 | 15.0 |
| divorced | 15.3 | 10.6 |
| widowed | 33.7 | 28.9 |
| Education |  |  |
| low | 26.0 | 15.8 |
| middle | 26.0 | 41.4 |
| high | 48.0 | 42.9 |

| **Risk / protective factor** | **Endorsed (%; total: n = 103)** | **Not endorsed (%; total: n = 372)** |
| --- | --- | --- |
| **Poor personal hygiene (sham-item)** |  |  |
| Age in years |  |  |
| 60-69 years | 21.0 | 37.9 |
| 70-79 years | 30.5 | 31.5 |
| 80-98 years | 48.6 | 30.7 |
| Women | 64.8 | 62.1 |
| Men | 35.2 | 37.9 |
| Marital status |  |  |
| married | 33.0 | 44.1 |
| single | 13.6 | 15.9 |
| divorced | 11.7 | 12.1 |
| widowed | 41.8 | 28.0 |
| Education |  |  |
| low | 22.3 | 17.5 |
| middle | 34.0 | 39.4 |
| high | 43.7 | 43.1 |

| **Risk / protective factor** | **Endorsed (%; total: n = 91)** | **Not endorsed (%; total: n = 342)** |
| --- | --- | --- |
| **Heart disease** |  |  |
| Age in years |  |  |
| 60-69 years | 34.1 | 36.3 |
| 70-79 years | 29.7 | 33.3 |
| 80-98 years | 36.3 | 30.4 |
| Women | 52.8 | 63.7 |
| Men | 47.3 | 36.3 |
| Marital status |  |  |
| married | 41.1 | 45.5 |
| single | 12.2 | 15.5 |
| divorced | 14.4 | 10.6 |
| widowed | 32.2 | 28.5 |
| Education |  |  |
| low | 17.6 | 19.7 |
| middle | 31.9 | 38.5 |
| high | 50.6 | 41.8 |

| **Risk / protective factor** | **Endorsed (%; total: n = 68)** | **Not endorsed (%; total: n = 334)** |
| --- | --- | --- |
| **Chronic kidney disease** |  |  |
| Age in years |  |  |
| 60-69 years | 25.0 | 38.2 |
| 70-79 years | 26.5 | 32.6 |
| 80-98 years | 48.5 | 29.0 |
| Women | 58.8 | 63.5 |
| Men | 41.2 | 36.5 |
| Marital status |  |  |
| married | 35.8 | 45.4 |
| single | 17.9 | 15.0 |
| divorced | 9.0 | 11.1 |
| widowed | 37.3 | 28.5 |
| Education |  |  |
| low | 16.2 | 18.1 |
| middle | 47.1 | 36.5 |
| high | 36.8 | 45.5 |

| **Risk / protective factor** | **Endorsed (%; total: n = 11)** | **Not endorsed (%; total: n = 472)** |
| --- | --- | --- |
| **Having children (sham-item)** |  |  |
| Age in years |  |  |
| 60-69 years | 36.4 | 33.7 |
| 70-79 years | 27.3 | 32.6 |
| 80-98 years | 36.4 | 33.7 |
| Women | 72.7 | 62.9 |
| Men | 27.3 | 37.1 |
| Marital status |  |  |
| married | 27.3 | 42.3 |
| single | 9.1 | 15.3 |
| divorced | 36.4 | 11.3 |
| widowed | 27.3 | 31.1 |
| Education |  |  |
| low | 9.1 | 18.9 |
| middle | 36.4 | 37.5 |
| high | 54.6 | 43.6 |

**Supplemantary Table 2: Sociodemographic characteristics and interest in information on brain health (“yes” / “maybe” vs. “no”)**

|  | **Interest in information on brain health (yes/maybe, %)** | **Interest in information on brain health (no, %)** |
| --- | --- | --- |
|  |  |  |
| Age in years |  |  |
| 60-69 years | 32.6 | 34.6 |
| 70-79 years | 31.1 | 32.7 |
| 80-98 years | 36.3 | 32.7 |
| Women | 58.4 | 65.7 |
| Men | 41.6 | 34.3 |
| Marital status |  |  |
| married | 47.6 | 38.0 |
| single | 14.3 | 15.9 |
| divorced | 11.6 | 11.7 |
| widowed | 26.5 | 34.4 |
| Education |  |  |
| low | 19.6 | 17.6 |
| middle | 30.7 | 41.0 |
| high | 49.7 | 41.4 |

**Supplemantary Table 3: Sociodemographic characteristics and openness towards eHealth interventions for brain health (“yes” / “maybe” vs. “no”)**

|  | **Openness towards eHealth (yes/maybe, %)** | **Openness towards eHealth (no, %)** |
| --- | --- | --- |
|  |  |  |
| Age in years |  |  |
| 60-69 years | 42.0 | 26.8 |
| 70-79 years | 33.3 | 30.9 |
| 80-98 years | 24.7 | 42.4 |
| Women | 60.6 | 64.7 |
| Men | 39.4 | 35.3 |
| Marital status |  |  |
| married | 48.3 | 35.8 |
| single | 14.8 | 16.0 |
| divorced | 12.6 | 10.8 |
| widowed | 24.4 | 37.3 |
| Education |  |  |
| low | 15.2 | 21.1 |
| middle | 34.6 | 39.5 |
| high | 50.2 | 39.5 |

**Supplementary Table 4: Factors associated with interest in information on brain health (univariable regression)**

|  | **Total sample** | | | **Age ≤ 75** | | |
| --- | --- | --- | --- | --- | --- | --- |
| **Variable** | **OR** | **SE** | **95% CI** | **OR** | **SE** | **95% CI** |
| Age in years | 1.01 | .01 | .99; 1.04 | 1.02 | .03 | .96; 1.08 |

|  | **Total sample** | | | **Age ≤ 75** | | |
| --- | --- | --- | --- | --- | --- | --- |
| **Variable** | **OR** | **SE** | **95% CI** | **OR** | **SE** | **95% CI** |
| Female (ref: male) | **.64** | .12 | .43; .96 | **.58** | .15 | .35; .96 |

|  | **Total sample** | | | **Age ≤ 75** | | |
| --- | --- | --- | --- | --- | --- | --- |
| **Variable** | **OR** | **SE** | **95% CI** | **OR** | **SE** | **95% CI** |
| Marital status (ref.: married) |  |  |  |  |  |  |
| Single | .66 | .19 | .38; 1.16 | .79 | .27 | .40; 1.54 |
| Divorced | .77 | .24 | .42; 1.43 | .57 | .24 | .24; 1.31 |
| Widowed | **.52** | .12 | .33; .82 | **.36** | .15 | .16; .79 |

|  | **Total sample** | | | **Age ≤ 75** | | |
| --- | --- | --- | --- | --- | --- | --- |
| **Variable** | **OR** | **SE** | **95% CI** | **OR** | **SE** | **95% CI** |
| Education (ref: low) |  |  |  |  |  |  |
| Moderate | .59 | .17 | .34; 1.03 | .49 | .20 | .22; 1.09 |
| High | .96 | .26 | .57; 1.63 | .82 | .32 | .38; 1.75 |

|  | **Total sample** | | | **Age ≤ 75** | | |
| --- | --- | --- | --- | --- | --- | --- |
| **Variable** | **OR** | **SE** | **95% CI** | **OR** | **SE** | **95% CI** |
| Subjective knowledge on dementia (ref: very much) |  |  |  |  |  |  |
| A lot | .97 | .29 | .54; 1.74 | 1.19 | .47 | .55; 2.59 |
| Something | 1.18 | .35 | .66; 2.11 | 1.28 | .51 | .58; 2.81 |
| Rather little | .73 | .31 | .31; 1.70 | .79 | .43 | .27; 2.32 |
| Nothing | .49 | .32 | .14; 1.78 |  |  |  |

|  | **Total sample** | | | **Age ≤ 75** | | |
| --- | --- | --- | --- | --- | --- | --- |
| **Variable** | **OR** | **SE** | **95% CI** | **OR** | **SE** | **95% CI** |
| Health literacy (HLS-EU-Q16; ref: inadequate) |  |  |  |  |  |  |
| Problematic | .43 | .18 | .19; 1.00 | .55 | .31 | .18; 1.66 |
| Sufficient | **.44** | .18 | .20; .97 | .62 | .31 | .23; 1.67 |

|  | **Total sample** | | | **Age ≤ 75** | | |
| --- | --- | --- | --- | --- | --- | --- |
| **Variable** | **OR** | **SE** | **95% CI** | **OR** | **SE** | **95% CI** |
| Resilience (BRS-Score) | .84 | .12 | .63; 1.12 | .82 | .16 | .56; 1.21 |

|  | **Total sample** | | | **Age ≤ 75** | | |
| --- | --- | --- | --- | --- | --- | --- |
| **Variable** | **OR** | **SE** | **95% CI** | **OR** | **SE** | **95% CI** |
| Knowledge of risk and protective factors (sum score) | **1.08** | .03 | 1.02; 1.15 | **1.08** | .04 | 1.00; 1.16 |

|  | **Total sample** | | | **Age ≤ 75** | | |
| --- | --- | --- | --- | --- | --- | --- |
| **Variable** | **OR** | **SE** | **95% CI** | **OR** | **SE** | **95% CI** |
| Knowing someone with dementia (ref.: no) | 1.22 | .26 | .81; 1.86 | 1.54 | .46 | .85; 2.78 |

|  | **Total sample** | | | **Age ≤ 75** | | |
| --- | --- | --- | --- | --- | --- | --- |
| **Variable** | **OR** | **SE** | **95% CI** | **OR** | **SE** | **95% CI** |
| Openness towards early diagnosis (ref.: no) | **1.89** | .40 | 1.25; 2.86 | **2.18** | .61 | 1.26; 3.77 |

**Supplementary Table 5: Factors associated with openness towards eHealth interventions for brain health (univariable regression)**

|  | **Total sample** | | | **Age ≤ 75** | | |
| --- | --- | --- | --- | --- | --- | --- |
| **Variable** | **OR** | **SE** | **95% CI** | **OR** | **SE** | **95% CI** |
| Age in years | .98 | .03 | .93; 1.04 | **.96** | .01 | .93; .97 |

|  | **Total sample** | | | **Age ≤ 75** | | |
| --- | --- | --- | --- | --- | --- | --- |
| **Variable** | **OR** | **SE** | **95% CI** | **OR** | **SE** | **95% CI** |
| Female (ref: male) | .84 | .16 | .58; 1.24 | .86 | .22 | .53; 1.41 |

|  | **Total sample** | | | **Age ≤ 75** | | |
| --- | --- | --- | --- | --- | --- | --- |
| **Variable** | **OR** | **SE** | **95% CI** | **OR** | **SE** | **95% CI** |
| Marital status (ref.: married) |  |  |  |  |  |  |
| Single | .67 | .19 | .39; 1.15 | .63 | .21 | .33; 1.23 |
| Divorced | .79 | .24 | .43; 1.44 | .50 | .20 | .23; 1.08 |
| Widowed | **.42** | .10 | .26; .65 | **.30** | .11 | .15; .62 |

|  | **Total sample** | | | **Age ≤ 75** | | |
| --- | --- | --- | --- | --- | --- | --- |
| **Variable** | **OR** | **SE** | **95% CI** | **OR** | **SE** | **95% CI** |
| Education (ref: low) |  |  |  |  |  |  |
| Moderate | 1.19 | .33 | .70; 2.05 | .99 | .39 | .46; 2.12 |
| High | **1.77** | .48 | 1.04; 3.00 | 1.88 | .73 | .88; 4.03 |

|  | **Total sample** | | | **Age ≤ 75** | | |
| --- | --- | --- | --- | --- | --- | --- |
| **Variable** | **OR** | **SE** | **95% CI** | **OR** | **SE** | **95% CI** |
| Subjective knowledge on dementia (ref: very much) |  |  |  |  |  |  |
| A lot | 1.31 | .38 | .74; 2.32 | 1.28 | .48 | .61; 2.68 |
| Something | 1.48 | .43 | .83; 2.62 | 1.45 | .56 | .68; 3.07 |
| Rather little | .64 | .25 | .30; 1.39 | .78 | .40 | .29; 2.12 |
| Nothing | .50 | .33 | .13; 1.84 | no obs. |  |  |

|  | **Total sample** | | | **Age ≤ 75** | | |
| --- | --- | --- | --- | --- | --- | --- |
| **Variable** | **OR** | **SE** | **95% CI** | **OR** | **SE** | **95% CI** |
| Health literacy (HLS-EU-Q16; ref: inadequate) |  |  |  |  |  |  |
| Problematic | .89 | .38 | .38; 2.06 | .77 | .43 | .25; 2.32 |
| Sufficient | 1.04 | .42 | .47; 2.31 | .81 | .42 | .30; 2.24 |

|  | **Total sample** | | | **Age ≤ 75** | | |
| --- | --- | --- | --- | --- | --- | --- |
| **Variable** | **OR** | **SE** | **95% CI** | **OR** | **SE** | **95% CI** |
| Resilience (BRS-Score) | .95 | .13 | .72; 1.26 | .93 | .18 | .64; 1.36 |

|  | **Total sample** | | | **Age ≤ 75** | | |
| --- | --- | --- | --- | --- | --- | --- |
| **Variable** | **OR** | **SE** | **95% CI** | **OR** | **SE** | **95% CI** |
| Knowledge of risk and protective factors (sum score) | **1.10** | .03 | 1.04; 1.17 | **1.13** | .04 | 1.04; 1.22 |

|  | **Total sample** | | | **Age ≤ 75** | | |
| --- | --- | --- | --- | --- | --- | --- |
| **Variable** | **OR** | **SE** | **95% CI** | **OR** | **SE** | **95% CI** |
| Knowing someone with dementia (ref.: no) | **1.83** | .39 | 1.21; 2.76 | **2.16** | .62 | 1.24; 3.78 |

|  | **Total sample** | | | **Age ≤ 75** | | |
| --- | --- | --- | --- | --- | --- | --- |
| **Variable** | **OR** | **SE** | **95% CI** | **OR** | **SE** | **95% CI** |
| Openness towards early diagnosis (ref.: no) | .99 | .01 | .97; 1.01 | 1.00 | .02 | .96; 1.04 |
